# Ethyl 3-amino-5-anilino-4-cyano­thio­phene-2-carboxyl­ate

**DOI:** 10.1107/S1600536813018734

**Published:** 2013-07-13

**Authors:** Ahmed M. M. El-Saghier, Mehmet Akkurt, Shaaban K. Mohamed, Peter N. Horton, Mustafa R. Albayati

**Affiliations:** a Chemistry Department, Faculty of Science, Sohag University, 82524 Sohag, Egypt; b Department of Physics, Faculty of Sciences, Erciyes University, 38039 Kayseri, Turkey; c Chemistry and Environmental Division, Manchester Metropolitan University, Manchester M1 5GD, England; d Chemistry Department, Faculty of Science, Mini University, 61519 El-Minia, Egypt; e School of Chemistry, University of Southampton, Highfield, Southampton SO17 1BJ, England; f Kirkuk University, College of Science, Department of Chemistry, Kirkuk, Iraq

## Abstract

In the title compound, C_14_H_13_N_3_O_2_S, the dihedral angle between the thio­phene and phenyl rings is 24.95 (8)°. The mol­ecular structure is consolidated by intra­molecular N—H⋯O and C—H⋯S inter­actions. The crystal structure features N—H⋯N and N—H⋯O hydrogen bonds forming centrosymmetric *R*_2_^2^(12) dimers, which are linked into a two-dimensional network parallel to (011) with an *S*(6)*R*_2_^2^ *S*(6) motif. In addition, π–π stacking inter­actions [centroid–centroid distance = 3.7013 (12) Å] occur between the thio­phene and phenyl rings of adjacent mol­ecules.

## Related literature

For pharmaceutical and industrial applications of amino-thio­phene-containingg compounds, see: Inversen *et al.* (2000 ▶); Webb *et al.* (2000 ▶). For the synthesis of multi-substituted thiphene compounds, see: El-Sharkawy *et al.* (2012 ▶); Huang *et al.* (2011 ▶). For the crystal structure of a related compound, see: Mabkhot *et al.* (2013 ▶).
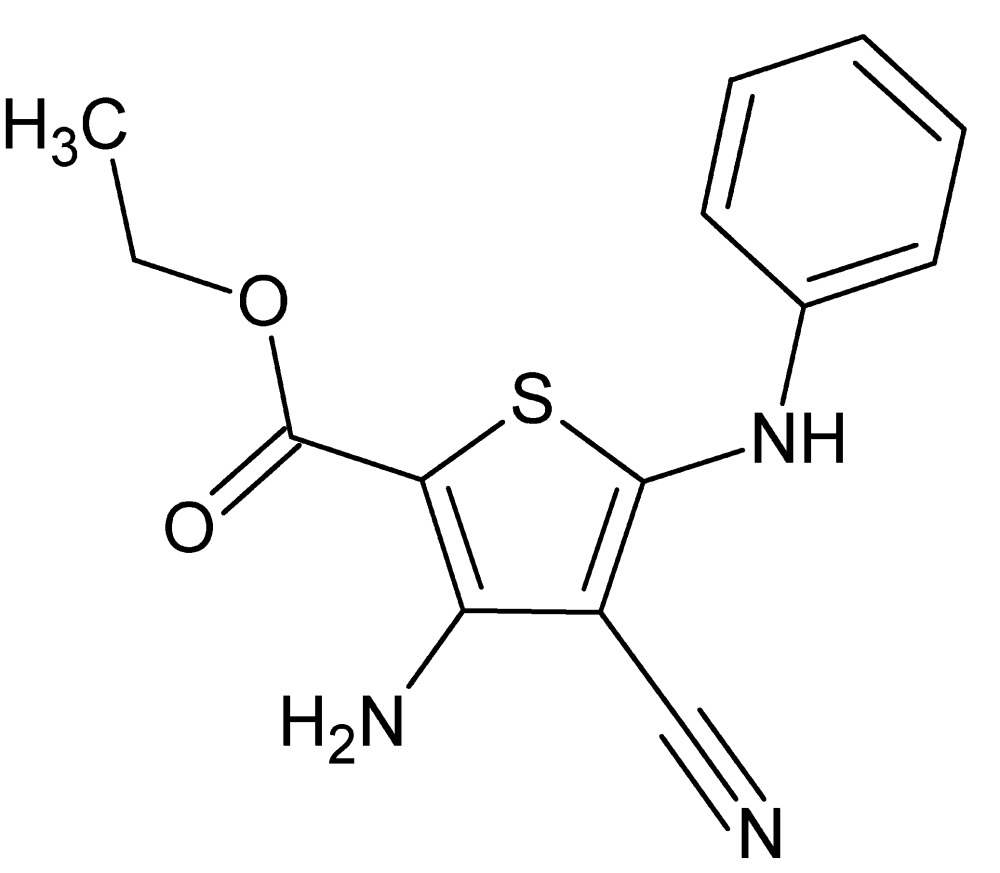


## Experimental

### 

#### Crystal data

C_14_H_13_N_3_O_2_S
*M* *_r_* = 287.34
Monoclinic, 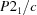

*a* = 8.6121 (15) Å
*b* = 10.6579 (15) Å
*c* = 14.328 (3) Å
β = 92.580 (3)°
*V* = 1313.8 (4) Å^3^
*Z* = 4
Mo *K*α radiation
μ = 0.25 mm^−1^
*T* = 100 K
0.55 × 0.04 × 0.03 mm

#### Data collection

Rigaku AFC12 (Right, Saturn724+) diffractometer
Absorption correction: multi-scan (*CrystalClear-SM Expert*; Rigaku, 2012 ▶) *T*_min_ = 0.887, *T*_max_ = 1.000
8995 measured reflections
2996 independent reflections
2728 reflections with *I* > 2σ(*I*)
*R*_int_ = 0.025

#### Refinement

*R*[*F* ^2^ > 2σ(*F* ^2^)] = 0.038
*wR*(*F* ^2^) = 0.088
*S* = 1.07
2996 reflections
194 parameters
H atoms treated by a mixture of independent and constrained refinement
Δρ_max_ = 0.32 e Å^−3^
Δρ_min_ = −0.26 e Å^−3^

### 

Data collection: *CrystalClear-SM Expert* (Rigaku, 2012 ▶); cell refinement: *CrystalClear-SM Expert*; data reduction: *CrystalClear-SM Expert*; program(s) used to solve structure: *SHELXS97* (Sheldrick, 2008 ▶); program(s) used to refine structure: *SHELXL97* (Sheldrick, 2008 ▶); molecular graphics: *ORTEP-3 for Windows* (Farrugia, 2012 ▶); software used to prepare material for publication: *WinGX* (Farrugia, 2012 ▶) and *PLATON* (Spek, 2009 ▶).

## Supplementary Material

Crystal structure: contains datablock(s) global, I. DOI: 10.1107/S1600536813018734/pv2639sup1.cif

Structure factors: contains datablock(s) I. DOI: 10.1107/S1600536813018734/pv2639Isup2.hkl

Supplementary material file. DOI: 10.1107/S1600536813018734/pv2639Isup3.cml

Additional supplementary materials:  crystallographic information; 3D view; checkCIF report

## Figures and Tables

**Table 1:** Hydrogen-bond geometry (Å, °)

*D*—H⋯*A*	*D*—H	H⋯*A*	*D*⋯*A*	*D*—H⋯*A*
N3—H3*NA*⋯O2	0.87 (2)	2.25 (2)	2.8671 (19)	128.0 (17)
N1—H1*N*⋯N2^i^	0.84 (2)	2.23 (2)	3.026 (2)	158.1 (16)
N3—H3*NB*⋯O2^ii^	0.86 (2)	2.24 (2)	3.0985 (18)	175.9 (17)
C10—H10⋯S1	0.95	2.55	3.1463 (17)	121

Symmetry codes: (i) 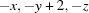; (ii) 
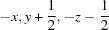
.

**Fig. 1.**
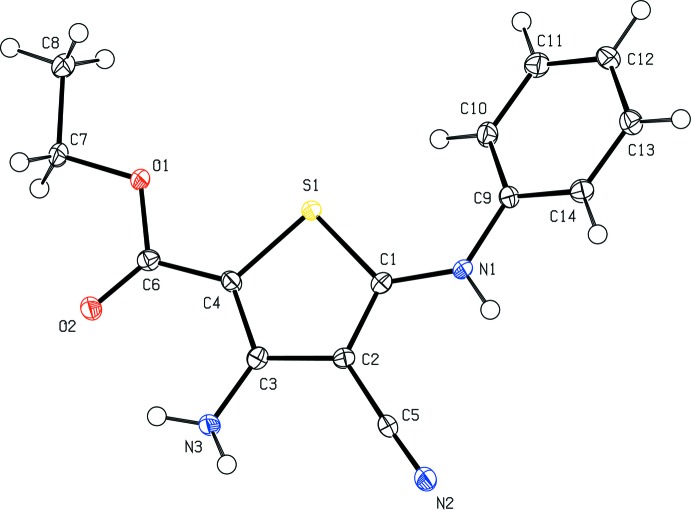
The molecular structure of the title compound with the atom numbering scheme. Displacement ellipsoids for non-H atoms are drawn at the 50% probability level.

**Fig. 2.**
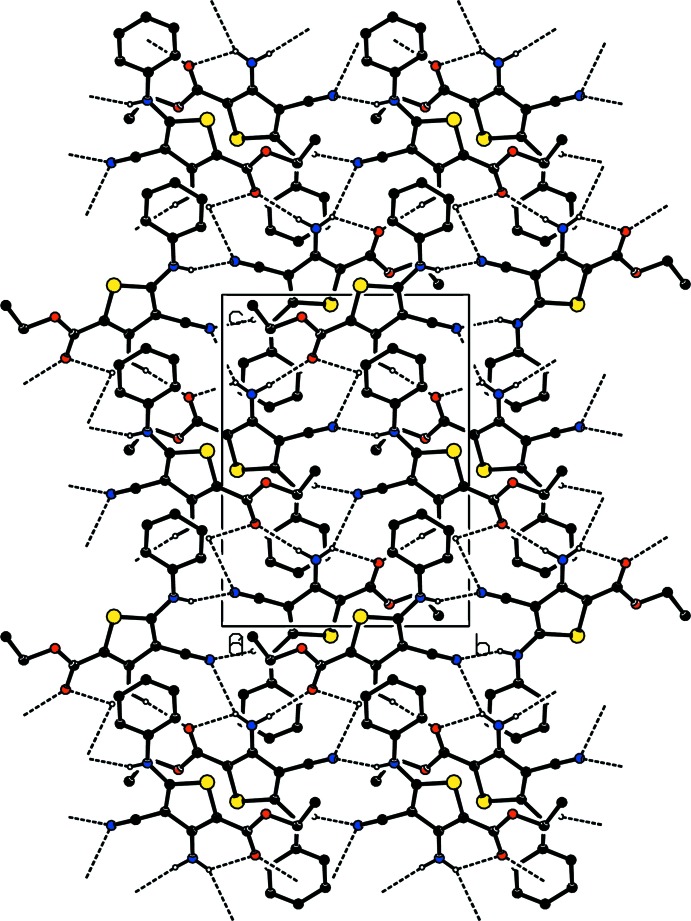
A view along the a axis of the packing diagram of the title compound showing hydrogen bonds as dashed lines. H atoms not involved in hydrogen bonds have been omitted for clarity.

**Fig. 3.**
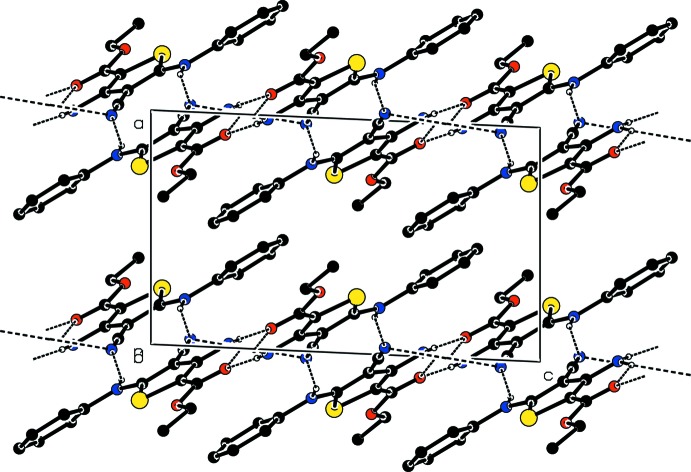
A view along the b axis of the packing diagram of the title compound showing hydrogen bonds as dashed lines. H atoms not involved in hydrogen bonds have been omitted for clarity.

## supplementary crystallographic information

### Comment

Multisubstituted thiophenes, particularly their amino derivatives which are widely used as active bio-molecules as inhibitors of several enzymes (Inversen *et al.*, 2000; Webb *et al.*, 2000). Moreover, carbonitril amino thiophen containingg compounds have been used as anticonvulsant and *CNS* antidepressant agents (El-Sharkawy *et al.*, 2012; Huang *et al.*, 2011). Based on such findings and further to our on going study in the synthesis of potential biologically active compouds, we herein report the synthesis and crystal structure of the title compound.

The bond distances and angles in the title compound (Fig. 1) agree very well with the corresponding bond distances and angles reported in a closely related compound (Mabkhot *et al.*, 2013). In the title molecule, the thiophene and phenyl rings make a dihedral angle of 24.95 (8)° with each other. The molecular structure is consolidated by intramolecular interactions N3—H3NA···O2 and C10—H10···S1 (Tab. 1). The crystal structure is stabilized by N1—H1N···N2 and N3—H3NB···O2 hydrogen bonds (Table 1, Figs. 2 & 3). Furthermore, π-π stacking interactions [*Cg*1···*Cg*2 (*x*, 3/2 - *y*, -1/2 + *z*) = 3.7013 (12) Å] between the centroids (*Cg*1 and *Cg*2, respectively) of the thiophene and phenyl rings of the consecutive molecules, contribute to the stabilization of the molecular packing of the title compound.

### Experimental

A solution of ethyl chloroacetate (54 ml, 5 mmol) in ethanol (20 ml) was added to a stirred solution of potassium 2,2-dicyano-1-(phenylamino)ethenethiolate (1.19 g, 5 mmol) in distilled water (20 ml) at room temperature. The reaction mixture was heated at 333 – 343 K for about 2 h. The precipitated ethyl 3-amino-4-cyano-5-(phenylamino)thiophene-2-carboxylate was was filtered off, dried and recrystallized from benzene to give high quality crystals (m.p.: 546–548 K) suitable for X-ray analysis in an excellent yield (76%).

### Refinement

The C-bound H atoms were placed geometrically with C—H = 0.95–0.99 Å and were refined using a riding model with *U*_iso_(H) = 1.2 or 1.5*U*_eq_(C). N-bound H atoms were located in a difference Fourier map and were refined freely.

### Crystal data

**Table:**

C_14_H_13_N_3_O_2_S	*F*(000) = 600
*M**_r_* = 287.34	*D*_x_ = 1.453 Mg m^−^^3^
Monoclinic, *P*2_1_/*c*	Mo *K*α radiation, λ = 0.71075 Å
Hall symbol: -P 2ybc	Cell parameters from 4552 reflections
*a* = 8.6121 (15) Å	θ = 2.4–30.2°
*b* = 10.6579 (15) Å	µ = 0.25 mm^−^^1^
*c* = 14.328 (3) Å	*T* = 100 K
β = 92.580 (3)°	Rod, light brown
*V* = 1313.8 (4) Å^3^	0.55 × 0.04 × 0.03 mm
*Z* = 4	

### Data collection

**Table:**

Rigaku AFC12 (Right, Saturn724+) diffractometer	2996 independent reflections
Radiation source: Rotating Anode	2728 reflections with *I* > 2σ(*I*)
Detector resolution: 28.5714 pixels mm^-1^	*R*_int_ = 0.025
profile data from ω–scans	θ_max_ = 27.5°, θ_min_ = 3.0°
Absorption correction: multi-scan (*CrystalClear-SM Expert*; Rigaku, 2012)	*h* = −7→11
*T*_min_ = 0.887, *T*_max_ = 1.000	*k* = −10→13
8995 measured reflections	*l* = −18→17

### Refinement

**Table:**

Refinement on *F*^2^	0 restraints
Least-squares matrix: full	Hydrogen site location: mixed
*R*[*F*^2^ > 2σ(*F*^2^)] = 0.038	H atoms treated by a mixture of independent and constrained refinement
*wR*(*F*^2^) = 0.088	W = 1/[Σ^2^(*F*O^2^) + (0.036*P*)^2^ + 0.9009*P*] WHERE *P* = (*F*O^2^ + 2*F*C^2^)/3
*S* = 1.07	(Δ/σ)_max_ = 0.001
2996 reflections	Δρ_max_ = 0.32 e Å^−^^3^
194 parameters	Δρ_min_ = −0.26 e Å^−^^3^

### Special details

**Table:**

Geometry. Bond distances, angles *etc*. have been calculated using the rounded fractional coordinates. All su's are estimated from the variances of the (full) variance-covariance matrix. The cell e.s.d.'s are taken into account in the estimation of distances, angles and torsion angles
Refinement. Refinement on *F*^2^ for ALL reflections except those flagged by the user for potential systematic errors. Weighted *R*-factors *wR* and all goodnesses of fit *S* are based on *F*^2^, conventional *R*-factors *R* are based on *F*, with *F* set to zero for negative *F*^2^. The observed criterion of *F*^2^ > σ(*F*^2^) is used only for calculating -*R*-factor-obs *etc*. and is not relevant to the choice of reflections for refinement. *R*-factors based on *F*^2^ are statistically about twice as large as those based on *F*, and *R*-factors based on ALL data will be even larger.

### Fractional atomic coordinates and isotropic or equivalent isotropic displacement parameters (Å^2^)

**Table:**

	*x*	*y*	*z*	*U*_iso_*/*U*_eq_	
S1	0.24094 (5)	0.56107 (3)	0.02889 (3)	0.0127 (1)	
O1	0.25546 (13)	0.32279 (10)	−0.06700 (7)	0.0146 (3)	
O2	0.09078 (14)	0.36573 (10)	−0.19090 (7)	0.0165 (3)	
N1	0.19895 (16)	0.80331 (12)	0.08703 (9)	0.0139 (4)	
N2	−0.03010 (18)	0.94889 (13)	−0.10133 (9)	0.0198 (4)	
N3	−0.01723 (17)	0.62007 (13)	−0.20023 (9)	0.0147 (4)	
C1	0.16973 (18)	0.71241 (14)	0.02243 (10)	0.0122 (4)	
C2	0.08009 (18)	0.73139 (14)	−0.05985 (10)	0.0128 (4)	
C3	0.06672 (18)	0.62244 (14)	−0.11815 (10)	0.0126 (4)	
C4	0.14784 (19)	0.52211 (14)	−0.07826 (10)	0.0130 (4)	
C5	0.01734 (19)	0.85067 (15)	−0.08401 (10)	0.0143 (4)	
C6	0.15918 (18)	0.39930 (14)	−0.11823 (10)	0.0129 (4)	
C7	0.2663 (2)	0.19549 (14)	−0.10206 (11)	0.0168 (4)	
C8	0.3668 (2)	0.12347 (15)	−0.03187 (11)	0.0199 (5)	
C9	0.28921 (18)	0.79947 (14)	0.17173 (10)	0.0130 (4)	
C10	0.3166 (2)	0.69024 (14)	0.22309 (11)	0.0162 (4)	
C11	0.4088 (2)	0.69608 (15)	0.30549 (11)	0.0191 (5)	
C12	0.4702 (2)	0.80873 (15)	0.33839 (11)	0.0176 (5)	
C13	0.43678 (19)	0.91814 (15)	0.28866 (11)	0.0164 (4)	
C14	0.34788 (19)	0.91355 (15)	0.20580 (11)	0.0153 (4)	
H3NA	−0.004 (2)	0.554 (2)	−0.2350 (14)	0.024 (5)*	
H1N	0.158 (2)	0.8734 (18)	0.0753 (12)	0.012 (4)*	
H3NB	−0.039 (2)	0.6900 (19)	−0.2278 (13)	0.018 (5)*	
H7A	0.31360	0.19520	−0.16380	0.0200*	
H7B	0.16180	0.15710	−0.10890	0.0200*	
H8A	0.47190	0.15900	−0.02890	0.0300*	
H8B	0.37140	0.03520	−0.05080	0.0300*	
H8C	0.32240	0.12940	0.02980	0.0300*	
H10	0.27300	0.61270	0.20230	0.0190*	
H11	0.43010	0.62130	0.33980	0.0230*	
H12	0.53430	0.81110	0.39410	0.0210*	
H13	0.47510	0.99640	0.31160	0.0200*	
H14	0.32660	0.98860	0.17180	0.0180*	

### Atomic displacement parameters (Å^2^)

**Table:**

	*U*^11^	*U*^22^	*U*^33^	*U*^12^	*U*^13^	*U*^23^
S1	0.0162 (2)	0.0097 (2)	0.0119 (2)	0.0022 (1)	−0.0026 (1)	−0.0003 (1)
O1	0.0197 (6)	0.0098 (5)	0.0139 (5)	0.0033 (4)	−0.0035 (4)	−0.0016 (4)
O2	0.0231 (6)	0.0123 (5)	0.0138 (5)	0.0014 (4)	−0.0039 (4)	−0.0009 (4)
N1	0.0181 (7)	0.0095 (6)	0.0136 (6)	0.0043 (5)	−0.0033 (5)	−0.0007 (5)
N2	0.0284 (8)	0.0151 (7)	0.0153 (7)	0.0043 (6)	−0.0062 (6)	−0.0022 (5)
N3	0.0196 (8)	0.0114 (7)	0.0128 (6)	0.0016 (5)	−0.0035 (5)	0.0004 (5)
C1	0.0126 (8)	0.0106 (7)	0.0136 (7)	0.0009 (6)	0.0017 (6)	0.0006 (5)
C2	0.0140 (8)	0.0116 (7)	0.0127 (7)	0.0011 (6)	−0.0001 (6)	0.0011 (5)
C3	0.0134 (8)	0.0110 (7)	0.0134 (7)	−0.0005 (6)	0.0019 (6)	0.0006 (6)
C4	0.0151 (8)	0.0128 (7)	0.0108 (7)	−0.0008 (6)	−0.0017 (6)	−0.0002 (5)
C5	0.0162 (8)	0.0157 (8)	0.0108 (7)	0.0011 (6)	−0.0015 (6)	−0.0026 (6)
C6	0.0138 (8)	0.0129 (7)	0.0121 (7)	0.0004 (6)	0.0010 (6)	0.0021 (5)
C7	0.0235 (9)	0.0106 (7)	0.0161 (7)	0.0037 (6)	−0.0022 (6)	−0.0026 (6)
C8	0.0262 (9)	0.0149 (8)	0.0184 (8)	0.0057 (7)	−0.0025 (7)	−0.0012 (6)
C9	0.0144 (8)	0.0142 (7)	0.0105 (7)	0.0016 (6)	0.0007 (6)	−0.0011 (6)
C10	0.0217 (9)	0.0121 (7)	0.0147 (7)	0.0010 (6)	−0.0001 (6)	−0.0013 (6)
C11	0.0264 (9)	0.0161 (8)	0.0148 (7)	0.0048 (7)	−0.0002 (7)	0.0021 (6)
C12	0.0193 (9)	0.0209 (8)	0.0123 (7)	0.0028 (6)	−0.0017 (6)	−0.0006 (6)
C13	0.0169 (8)	0.0158 (8)	0.0164 (7)	−0.0016 (6)	−0.0003 (6)	−0.0014 (6)
C14	0.0183 (8)	0.0128 (7)	0.0147 (7)	0.0004 (6)	0.0003 (6)	0.0019 (6)

### Geometric parameters (Å, º)

**Table:**

S1—C1	1.7266 (16)	C7—C8	1.507 (2)
S1—C4	1.7497 (16)	C9—C14	1.396 (2)
O1—C6	1.3552 (19)	C9—C10	1.392 (2)
O1—C7	1.4512 (19)	C10—C11	1.394 (2)
O2—C6	1.2264 (18)	C11—C12	1.386 (2)
N1—C1	1.356 (2)	C12—C13	1.390 (2)
N1—C9	1.412 (2)	C13—C14	1.384 (2)
N2—C5	1.147 (2)	C7—H7A	0.9900
N3—C3	1.353 (2)	C7—H7B	0.9900
N1—H1N	0.84 (2)	C8—H8A	0.9800
N3—H3NB	0.86 (2)	C8—H8B	0.9800
N3—H3NA	0.87 (2)	C8—H8C	0.9800
C1—C2	1.395 (2)	C10—H10	0.9500
C2—C3	1.432 (2)	C11—H11	0.9500
C2—C5	1.418 (2)	C12—H12	0.9500
C3—C4	1.386 (2)	C13—H13	0.9500
C4—C6	1.434 (2)	C14—H14	0.9500
			
C1—S1—C4	91.55 (7)	N1—C9—C14	116.86 (13)
C6—O1—C7	114.99 (11)	C9—C10—C11	119.14 (14)
C1—N1—C9	130.11 (13)	C10—C11—C12	121.38 (15)
C1—N1—H1N	115.8 (12)	C11—C12—C13	119.04 (15)
C9—N1—H1N	114.1 (12)	C12—C13—C14	120.25 (15)
C3—N3—H3NA	115.7 (13)	C9—C14—C13	120.54 (14)
H3NA—N3—H3NB	117.9 (19)	O1—C7—H7A	110.00
C3—N3—H3NB	118.7 (13)	O1—C7—H7B	110.00
S1—C1—C2	111.26 (11)	C8—C7—H7A	110.00
N1—C1—C2	123.52 (14)	C8—C7—H7B	110.00
S1—C1—N1	125.20 (11)	H7A—C7—H7B	109.00
C1—C2—C5	121.79 (14)	C7—C8—H8A	109.00
C3—C2—C5	124.39 (13)	C7—C8—H8B	109.00
C1—C2—C3	113.73 (13)	C7—C8—H8C	109.00
C2—C3—C4	111.05 (13)	H8A—C8—H8B	110.00
N3—C3—C2	123.23 (14)	H8A—C8—H8C	109.00
N3—C3—C4	125.71 (14)	H8B—C8—H8C	109.00
S1—C4—C3	112.41 (11)	C9—C10—H10	120.00
S1—C4—C6	122.03 (11)	C11—C10—H10	120.00
C3—C4—C6	125.56 (14)	C10—C11—H11	119.00
N2—C5—C2	177.72 (16)	C12—C11—H11	119.00
O2—C6—C4	124.52 (14)	C11—C12—H12	120.00
O1—C6—C4	112.58 (12)	C13—C12—H12	120.00
O1—C6—O2	122.90 (13)	C12—C13—H13	120.00
O1—C7—C8	106.81 (12)	C14—C13—H13	120.00
N1—C9—C10	123.53 (14)	C9—C14—H14	120.00
C10—C9—C14	119.57 (14)	C13—C14—H14	120.00
			
C4—S1—C1—N1	−179.31 (14)	C1—C2—C3—C4	−0.1 (2)
C4—S1—C1—C2	−0.86 (12)	N3—C3—C4—C6	1.4 (3)
C1—S1—C4—C6	−179.95 (13)	N3—C3—C4—S1	−179.43 (13)
C1—S1—C4—C3	0.82 (13)	C2—C3—C4—S1	−0.55 (17)
C7—O1—C6—O2	−2.8 (2)	C2—C3—C4—C6	−179.75 (15)
C7—O1—C6—C4	177.50 (13)	C3—C4—C6—O1	175.42 (14)
C6—O1—C7—C8	−175.31 (13)	C3—C4—C6—O2	−4.3 (3)
C9—N1—C1—C2	−178.43 (15)	S1—C4—C6—O1	−3.71 (19)
C9—N1—C1—S1	−0.2 (2)	S1—C4—C6—O2	176.63 (13)
C1—N1—C9—C14	155.95 (16)	N1—C9—C10—C11	179.34 (15)
C1—N1—C9—C10	−26.5 (3)	C14—C9—C10—C11	−3.1 (2)
N1—C1—C2—C3	179.20 (14)	N1—C9—C14—C13	179.64 (14)
S1—C1—C2—C5	−175.79 (12)	C10—C9—C14—C13	2.0 (2)
N1—C1—C2—C5	2.7 (2)	C9—C10—C11—C12	1.7 (3)
S1—C1—C2—C3	0.72 (17)	C10—C11—C12—C13	1.0 (3)
C5—C2—C3—C4	176.30 (15)	C11—C12—C13—C14	−2.2 (2)
C1—C2—C3—N3	178.81 (14)	C12—C13—C14—C9	0.8 (2)
C5—C2—C3—N3	−4.8 (2)		

### Hydrogen-bond geometry (Å, º)

**Table:**

*D*—H···*A*	*D*—H	H···*A*	*D*···*A*	*D*—H···*A*
N3—H3*NA*···O2	0.87 (2)	2.25 (2)	2.8671 (19)	128.0 (17)
N1—H1*N*···N2^i^	0.84 (2)	2.23 (2)	3.026 (2)	158.1 (16)
N3—H3*NB*···O2^ii^	0.86 (2)	2.24 (2)	3.0985 (18)	175.9 (17)
C10—H10···S1	0.95	2.55	3.1463 (17)	121

Symmetry codes: (i) −*x*, −*y*+2, −*z*; (ii) −*x*, *y*+1/2, −*z*−1/2.
